# Comparison of 3D Confromal Radiotherapy and Intensity Modulated Radiotherapy with or without Simultaneous Integrated Boost during Concurrent Chemoradiation for Locally Advanced Head and Neck Cancers

**DOI:** 10.1371/journal.pone.0094456

**Published:** 2014-04-08

**Authors:** Michael T. Spiotto, Ralph R. Weichselbaum

**Affiliations:** 1 Department of Radiation and Cellular Oncology, University of Chicago Medical Center, Chicago, Illinois, United States of America; 2 Department of Radiation Oncology, University of Illinois Hospital and Health Sciences System, Chicago, Illinois, United States of America; NIH, United States of America

## Abstract

**Objective:**

Radiotherapy techniques have evolved from 3D conformal radiotherapy (3D-CRT) to intensity modulated radiotherapy (IMRT) where boost fields are delivered either sequentially (IMRTseq) or with a simultaneous integrated boost (IMRT+SIB). Our goal was to compare the outcomes of patients treated with IMRT+SIB to traditional standards.

**Methods:**

We analyzed the efficacy and toxicity of patients treated with concurrent chemoradiation using 3D-CRT, IMRTseq or IMRT+SIB. Between 1993 and 2012, 379 patients with non-metastatic Stage III-IV head and neck squamous cell cancer were treated with concurrent chemoradiation using 3D-CRT (n = 125), IMRTseq (n = 120) and IMRT+SIB (n = 134).

**Results:**

Patients treated with any technique had similar rates of 2y local control, 2y regional control, 2y progression free survival and 2y overall survival. Patients treated with IMRT+SIB had lower rates acute toxicity according to Grade 3 or greater mucositis (3D-CRT: 44.0% vs. IMRTseq: 36.7% vs. IMRT+SIB: 22.4%; *P*<.0001), dermatitis (3D-CRT: 44.0% vs. IMRTseq: 20.0% vs. IMRT+SIB: 7.5%; *P*<.0001) and feeding tube placement during radiotherapy (3D-CRT: 80.0% vs. IMRTseq: 50.8% vs. IMRT+SIB: 44.0%; *P*<.0001) as well as late toxicity as measured by feeding tube use (*P*<.0001) and tracheostomy use (*P*<.0001). On multivariate analysis, IMRT+SIB predicted for less mucositis, dermatitis and feeding tube use compared to 3D-CRT and for less dermatitis compared to IMRTseq.

**Conclusions:**

Compared to 3D-CRT and IMRTseq, IMRT+SIB provided similar outcomes and potentially less toxicity indicating it is a feasible technique for chemoradiation in locally advanced head and neck cancer.

## Introduction

Over the last 20 years, the delivery of radiotherapy to different cancer sites has changed dramatically, especially for squamous cell carcinomas of the Head and Neck (HNSCCs) region [1]–[4]. HNSCC patients were classically treated with three dimensional conformal radiotherapy (3D-CRT) where increasing radiation doses were delivered to higher risk areas of disease using sequential radiotherapy plans to treat smaller boost fields, which was also known as a “shrinking-field approach”. Patients often experienced severe acute and late toxicities including mucositis, dermatitis and xerostomia. As intensity modulated radiotherapy (IMRT) was implemented, the improved high-dose conformality reduced the dose to normal tissues and offered the potential to minimize radiation toxicity while maintaining similar rates of control [2], [4]–[6]. Still, IMRT plans often relied on this sequential treatment design (IMRTseq). Consequently, using IMRTseq and 3D-CRT plans, each risk area received the same dose per fraction that usually did not exceed 2 Gy in order to minimize acute toxicity.

By contrast, IMRT treatment planning enables the simultaneous delivery of individualized dose levels to distinct at risk areas within a single treatment fraction [1], [3]. The advantages to IMRT+SIB includes increased planning efficiency and decreased planning uncertainty as radiation dose can be accounted for in a single plan. In addition, IMRT+SIB, escalates the dose per fraction delivered to the gross disease in order to potentially improve tumor control. Several groups have reported their single and multi-institutional experience in using IMRT+SIB [7]–[12]. However, it remains unclear how IMRT+SIB compares to previous radiation planning techniques such as IMRTseq or 3D-CRT when treating advanced HNSCC patients with chemoradiation. First, the increased dose per fraction may unintentionally increase acute toxicity as has been described in other altered fractionation series [13], [14]. When coupled with concurrent chemotherapy, the added toxicity may result in chemotherapy dose modifications that impact treatment efficacy [15]. By contrast, the decreased fractional dose delivered to lower risk nodal groups may also not be sufficient to control microscopic disease. Therefore, it remains unclear the extent to which the efficacy and toxicity IMRT+SIB compares to traditionally accepted radiotherapy planning techniques.

Our institution has employed all three techniques for the irradiation of locally advanced HNSCC. To this end, we compared the outcomes in patients treated with concurrent chemoradiation delivered using 3D-CRT, sequential IMRTseq or IMRT+SIB radiotherapy plans.

## Patients and Methods

### Study Population

We utilized a retrospective database of 803 patients with HNSCC to select 398 patients with Stage III-IVB disease treated with concurrent chemoradiation. 405 of 803 patients were not analyzed due to lower stage, non-concurrent chemotherapy usage or inadequate records. We excluded 19 patients who did not have adequate radiotherapy records and were excluded to give a total of 379 patients for analysis. Patients were treated at the University of Illinois Medical Center at Chicago between 1993 to 2012. This study was specifically approved by the University of Illinois Medical Center Institutional Review Board under protocol 2011-1075 in accordance with the ethical standards of the responsible committee on human experimentation and with the Helsinki Declaration of 1975, as revised in 2000. The University of Illinois at Chicago Institutional Review board waived informed consent given that this study used preexisting medical records and obtaining informed consent on all patients would be impractical given the associated time and cost.

### Treatment

All patients were treated with concurrent chemotherapy consisting Platin-containing regimens or paclitaxel, hydroxyurea and 5-flurouracil. Platin-containing regimens consisted of cisplatin or carboplatin given weekly or every three weeks. Of the 379 patients analyzed, 198 patients received concurrent Platin of which 146 patients received cisplatin and 52 patients received carboplatin, 137 patients received paclitaxel, hydroxyurea and 5-flurouracil and 44 patients did not have detailed chemotherapy records. 125 patients received 3D conformal radiotherapy (3D-CRT), 134 patients received IMRT+SIB and 120 patients received IMRTseq. For IMRT planning, the clinical target volumes were typically expanded by 5 mm margins to generate the planning target volumes (PTV). PTVs were shaved at the skin and near critical organs when radiotherapy plans exceeded normal tissue tolerance. In 82% of the patients treated with IMRT+SIB, the fractionation schemes consisted of either: (1) 30 fractions delivering 6600 cGy to the GTV, 6000 to the intermediate risk nodal volumes and 5400 cGy to the low risk nodal volumes or (2) 33 fractions delivering 6996 cGy to the GTV, 5940 cGy to the intermediate risk nodal volumes and 5412 cGy to the low risk nodal volumes. Patients treated with IMRTseq or 3D-CRT were treated with daily fractionation in 200 cGy fractions or twice daily fractionation in 120–150 cGy fractions. Weekly megavoltage portal films were used for localization. No image guided radiotherapy was used in any patients in order to decreased planning margins. During radiotherapy, acute toxicities were recorded during weekly on-treatment visits.

### Variables

We approximated comorbidity burden using a modified Charlson Comorbidity Index [16] and performance status using the Karnofsky Performance Status [17]. Staging was categorized using the American Joint Committee staging system at the time of diagnosis. Acute toxicity was scored using RTOG common toxicity criteria. We defined a truncated radiotherapy (RT) course as one shortened by more than 5 treatment fractions due to non-compliance. We defined RT delay as RT courses that were completed 5d or longer than the anticipated. We defined dose modifications during chemoradiation as reducing the drug dosage, switching from chemotherapy delivered every three weeks to weekly, delaying the intended chemotherapy cycle or holding chemotherapy during radiation. We divided the era of RT into three groups: (1) 1993–2000 when all patients were treated with 3D-CRT, (2) 2001–2006 when patients were predominantly treated with IMRTseq and (3) 2007–2012 when patients were predominantly treated with IMRT+SIB. Time to local control (LC), regional control (RC), progression free survival (PFS), and overall survival (OS) were determined from last date of RT. Patterns of local or regional failure were determined as the first failure with any component of local or regional failure, respectively. PFS was calculated as the time to any failure or death from any cause. OS was calculated as the time to death from any cause.

### Statistical analysis

Statistical analysis was performed using JMP version 9 (SAS Institute). All tests to determine statistical significance were two-sided and statistical significance was defined as *P*<.05. Discrete variables were compared with chi-square test and continuous variables were compared with the t-test. Differences between medians were assessed using the Wilcoxon test. Survival curves were plotted based on the Kaplan-Meier method. For univariate analysis of toxicity, we selected factors that were significantly different between the 3D-CRT, IMRTseq and IMRT+SIB groups. Multivariate analysis of toxicity was performed using nominal logistic regression analysis to adjust for explanatory confounding prognostic variables with *P* value<.1 on univariate analysis.

## Results

### Population and Tumor Characteristics

As shown in Table 1, median follow-up did not differ significantly between groups (25.8 mo 3D-CRT for vs. 17.5 mo for IMRTseq vs. 16.3 mo for IMRT+SIB; *P* = .14). Compared to IMRTseq based techniques, patients treated with 3D-CRT were younger (54.6y for 3D-CRT vs. 58.4y for IMRTseq vs. 59.1y for IMRT+SIB; P = .003) and had more alcohol use (70.4% for 3D-CRT vs. 54.2% for IMRTseq vs. 56.7% for IMRT+SIB; *P* = .03). Patients treated with 3D-CRT or IMRT+SIB had a greater smoking history compared to patients treated with IMRTseq (81.6% for 3D-CRT vs. 69.2% for IMRTseq vs. 79.9% for IMRT+SIB; *P* = .02). The three groups did not differ significantly based on gender, performance status, comorbidities, stage or primary site (Table1).

**Table 1:** Patient and Tumor Characteristics (n = 379).

Category	Indicator	3D-CRT^1^ (n = 125)	IMRTseq^2^ (n = 120)	IMRT+SIB^3^ (n = 134)	*P value*
Median age (years)		54.6	58.4	59.1	.003
	(IQR^4^)	(45.8–60.6)	(50.0–63.5)	(50.8–65.7)	
Median follow-up (months)		25.8	17.5	16.3	.14
	(IQR)	(6.4–68.6)	(7.4–47.1)	(8.0–39.1)	
Gender	Male	90 (72.0%)	91 (75.8%)	103 (76.9%)	.64
	Female	35 (28.0%)	29 (24.2%)	31 (23.1%)	
KPS^5^	<70	8 (6.4%)	6 (5.0%)	12 (9.0%)	.78
	≥70	102 (81.6%)	101 (84.2%)	106 (79.1%)	
	Not reported	15 (11.3%)	13 (10.8%)	16 (11.9%)	
Comorbidity	Medium	93 (77.4%)	78 (65.0%)	87 (64.9%)	.17
	High	32 (25.6%)	43 (35.0%)	47 (35.1%)	
T stage	Tx	3 (2.4%)	3 (2.5%)	6 (4.5%)	.29
	T1	4 (3.2%)	9 (7.5%)	8 (6.0%)	
	T2	14 (11.2%)	22 (18.3%)	23 (17.2%)	
	T3	34 (27.2%)	32 (26.7%)	41 (30.6%)	
	T4	14 (11.2%)	7 (5.8%)	5 (3.7%)	
	T4a	52 (41.6%)	44 (36.7%)	46 (34.3%)	
	T4b	4 (3.2%)	3 (2.5%)	5 (3.7%)	
N stage	N0	27 (21.6%)	27 (22.5%)	28 (20.9%)	.36
	N1	17 (13.6%)	13 (10.8%)	20 (14.9%)	
	N2	3 (2.4%)	5 (4.2%)	3 (2.2%)	
	N2a	1 (0.8%)	8 (6.7%)	7 (5.2%)	
	N2b	27 (21.6%)	24 (20.0%)	34 (25.4%)	
	N2c	28 (22.4%)	30 (25.0%)	26 (19.4%)	
	N3	22 (17.6%)	12 (10.0%)	15 (11.2%)	
	N.R.	1 (1.9%)	1 (0.8%)	1 (0.7%)	
Stage	III	19 (15.2%)	21 (17.5%)	32 (23.9%)	.12
	IVA	79 (63.2%)	84 (70.0%)	85 (63.4%)	
	IVB	27 (21.6%)	15 (12.5%)	17 (12.7%)	
Primary site	Hypopharynx	17 (14.3%)	7 (5.9%)	8 (6.1%)	.10
	Larynx	18 (15.1%)	24 (20.3%)	26 (19.7%)	
	Nasopharynx	9 (7.7%)	6 (5.1%)	6 (4.6%)	
	Oral cavity	32 (26.9%)	28 (23.7%)	39 (29.6%)	
	Oropharynx	35 (29.4%)	46 (39.0%)	43 (32.6%)	
	Unknown	3 (2.5%)	2 (1.7%)	6 (4.6%)	
	Other	11 (9.2%)	7 (5.9%)	6 (4.6%)	
Alcohol history	≥2 drinks/day	88 (70.4%)	65 (54.2%)	76 (56.7%)	.03
	<2 drinks/day	19 (15.2%)	27 (22.5%)	37 (27.6%)	
	Not reported	18 (14.4%)	28 (23.3%)	21 (15.7%)	
Tobacco history	Yes	102 (81.6%)	83 (69.2%)	107 (79.9%)	.02
	No	18 (14.4%)	32 (26.7%)	20 (14.9%)	
	Not reported	5 (4.2%)	5 (4.2%)	7 (5.2%)	

1 3D-CRT  =  Three dimensional conformal radiotherapy.

2 IMRTseq  =  Sequential intensity modulated radiotherapy.

3 IMRT+SIB  =  Intensity modulated radiotherapy with simultaneous integrated boost.

4 IQR  =  interquartile ratio.

5 KPS  =  Karnofsky.

### Treatment Characteristics

As shown in Table 2, more patients treated with IMRTseq received induction chemotherapy compared to patients treated with 3D-CRT or IMRT+SIB (32.8% for 3D-CRT vs. 50.8% for IMRTseq vs. 34.2% for IMRT+SIB; *P* = .006). Compared to 3D-CRT, patients treated with either IMRT technique received more platin-based concurrent chemoradiation (*P*<.0001) as well as experienced more modifications in concurrent chemotherapy. Patients treated with 3D-CRT received higher total radiation doses delivered to the gross tumor (7400 cGy for 3D-CRT vs. 7125cGy for IMRTseq vs. 6600 cGy for IMRT+SIB; *P*<.0001) but received lower total radiation dose to the intermediate and low risk nodal groups. Treatment technique varied according to the era of RT as patients treated before 2000 received 3D-CRT and the majority of patients treated after 2000 received IMRTseq or IMRT+SIB (*P*<.0001). Patients did not differ based on rates of post-operative RT or post-RT lymph node dissection.

**Table 2:** Treatment Characteristics (n = 379).

Category	Indicator	3D-CRT^1^ (n = 125)	IMRTseq^2^ (n = 120)	IMRT+SIB^3^ (n = 134)	*P value*
Induction chemotherapy	Yes	41 (32.8%)	61 (50.8%)	46 (34.2%)	.006
	No	84 (67.2%)	59 (49.2%)	88 (65.7%)	
Post-operative RT^4^	Yes	23 (18.4%)	30 (25.0%)	37 (27.6%)	.20
	No	102 (81.6%)	90 (75.0%)	97 (72.4%)	
Post-RT lymphadenectomy	Yes	27 (26.5%)	28 (31.1%)	20 (20.6%)	.26
	No	75 (73.5%)	62 (68.9%)	77 (79.4%)	
CTX^5^ dose modification	Yes	33 (26.4%)	52 (43.3%)	75 (56.0%)	<.0001
	No	92 (73.6%)	68 (56.7%)	59 (44.0%)	
Platin-based chemoradiation	Yes	31 (24.8%)	63 (52.5%)	104 (77.6%)	<.0001
	No	94 (75.2%)	57 (47.5%)	30 (22.4%)	
Median dose to gross tumor (cGy)		7400	7125	6600	<.0001
	IQR^6^	(7015–7450)	(6650–7250)	(6600–6996)	
Median dose to intermediate risk lymph nodes (cGy)		5200	5100	5940	<.0001
	IQR	(5045–5450)	(5000–5400)	(5940–6000)	
Median dose to low risk lymph nodes (cGy)		3950	5000	5400	<.0001
	IQR	(3900–5000)	(3650–5000)	(5400–5412)	
Alterations in RT course	No	70 (56.0%)	76 (63.3%)	66 (49.3%)	.06
	Delayed	36 (28.8%)	35 (29.2%)	43 (32.1%)	
	Truncated	19 (15.2%)	9 (7.5%)	25 (18.7%)	
Era of RT	1993–2000	90 (100.0%)	0 (0.0%)	0 (0.0%)	.06
	2001–2006	32 (22.4%)	84 (58.7%)	27 (18.9%)	
	2007–2012	3 (2.4%)	36 (24.7%)	107 (73.3%)	

1 3D-CRT  =  Three dimensional conformal radiotherapy.

2 IMRTseq  =  Sequential intensity modulated radiotherapy.

3 IMRT+SIB  =  Intensity modulated radiotherapy with simultaneous integrated boost.

4 RT  =  radiotherapy.

5 CTX  =  chemotherapy.

6 IQR  =  interquartile ratio.

### Outcomes

**Figure 1:**
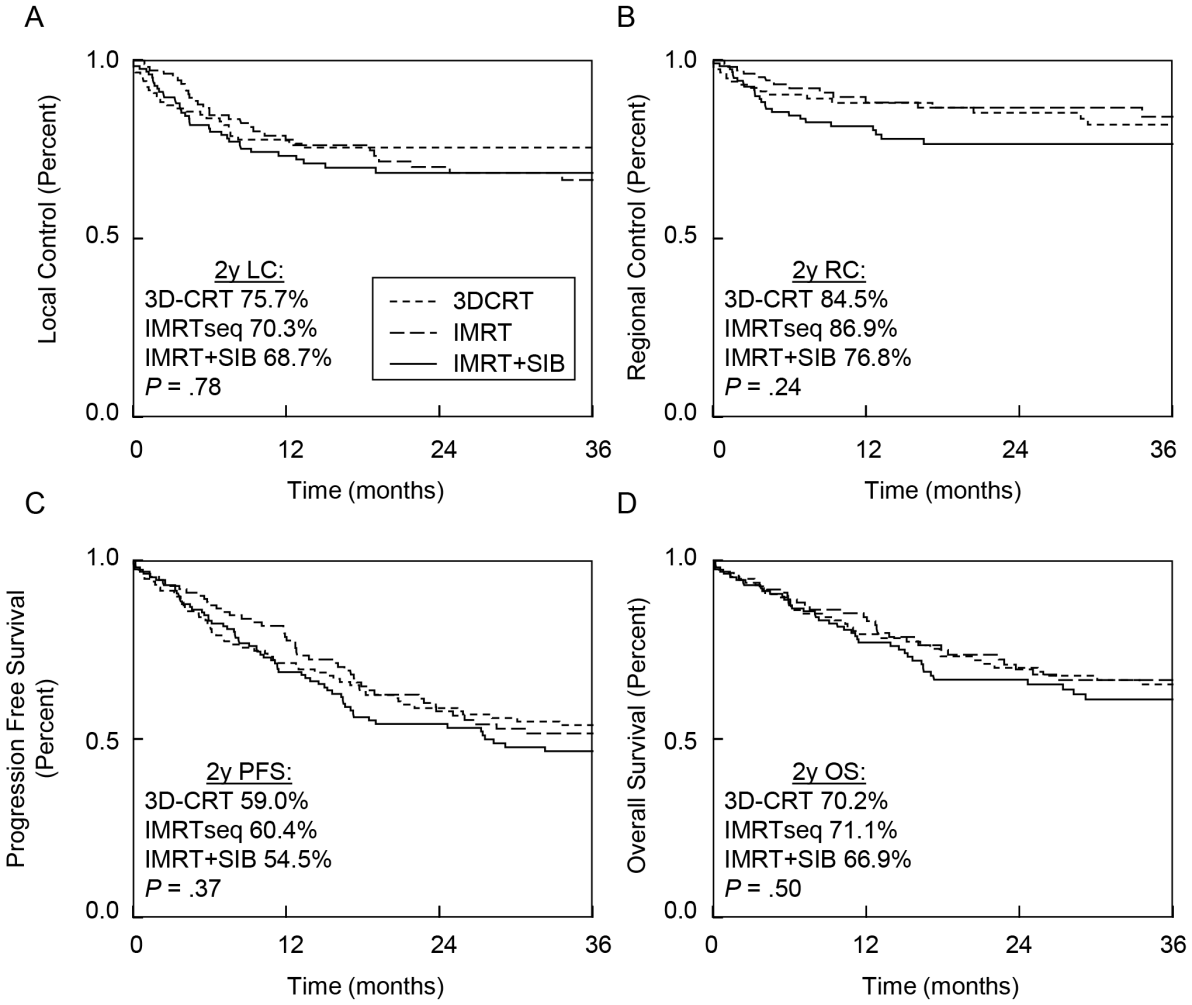
Kaplan-Meier analysis of outcomes in patients treated with 3D-CRT, IMRTseq and IMRT+SIB. (**a**) Local control (**b**) regional control (**c**) progression free survival and (**d**) overall survival for Stage III-IV HNSCC patients treated with chemoradiation using 3D-CRT, IMRTseq and IMRT+SIB. The log rank test was used to assess for differences in outcomes.

Compared to 3D-CRT, patients treated with IMRT+SIB had similar LC (HR 1.18; 95% CI 0.72–1.93; *P* = .51), RC (HR 1.41; 95% CI 0.77–2.60; *P* = .26), PFS (HR 1.21; 95% CI 0.87–1.70; *P* = .26) and OS (HR 1.19; 95% CI 0.79–1.79; *P* = .41). Similarly, compared to IMRTseq, patients treated with IMRT+SIB had similar LC (HR 1.15; 95% CI 0.70–1.89; *P* = .59), RC (HR 1.70; 95% CI 0.90–3.31; *P* = .10), PFS (HR 1.26; 95% CI 0.89–1.79; *P* = .20) and OS (HR 1.28; 95% CI 0.84–1.98; *P* = .26). As shown in Figure 1, 3D-CRT, IMRTseq and IMRT+SIB has similar rates of 2y LC (*P* = .78), 2y RC (*P* = .24), 2y PFS (*P* = .37) and 2y OS (*P* = .50).

### Toxicity

As shown in Table 3, patients treated with IMRT+SIB had lower rates of Grade ≥3 mucositis, Grade ≥3 dermatitis, feeding tube placement during RT as well as long term use of feeding tubes or tracheostomies (*P*<.0001). On univariate analysis, IMRT+SIB was associated with less mucositis compared to 3D-CRT (HR 0.29; 95% CI 0.16–0.50; *P*<.0001) or IMRTseq (HR 0.40; 95% CI 0.23–0.71; *P* = .002) as well as less dermatitis compared to 3D-CRT (HR 0.08; 95% CI 0.04–0.17; *P*<.0001) or IMRTseq (HR 0.28; 95% CI 0.12–0.60; *P* = .001). Compared to 3D-CRT, IMRT+SIB was associated with less feeding tube during RT (HR 0.26; 95% CI 0.14–0.45; *P*<.0001), long term feeding tube use (HR 0.24; 95% CI 0.14–0.41; *P*<.0001) and long term tracheostomy use (HR 0.26; 95% CI 0.14–0.47; *P*<.0001). By contrast, there was no difference between IMRTseq and IMRT+SIB for feeding tube placement during RT, long term feeding tube use or long term tracheostomy.

**Table 3:** Toxicity (n = 379).

Category	Indicator	3D-CRT^1^ (n = 125)	IMRTseq^2^ (n = 120)	IMRT+SIB^3^ (n = 134)	*P value*
Feeding tube during RT^4^	Yes	100 (80.0%)	61 (50.8%)	59 (44.0%)	<.0001
	No	25 (20.0%)	59 (49.2%)	75 (56.0%)	
Greater than 10% weight loss during RT	Yes	48 (38.4%)	64 (53.3%)	70 (52.2%)	.10
	No	38 (30.4%)	26 (21.7%)	43 (32.1%)	
	N.S. stated	39 (31.2%)	30 (25.0%)	30 (22.4%)	
Grade ≥3 mucositis	Yes	55 (44.0%)	44 (36.7%)	30 (22.4%)	<.0001
	No	48 (38.4%)	54 (45.0%)	91 (67.9%)	
	N.S. stated	22 (17.6%)	22 (18.3%)	13 (9.7%)	
Grade ≥3 dermatitis	Yes	55 (44.0%)	24 (20.0%)	10 (7.5%)	<.0001
	No	51 (40.8%)	74 (61.7%)	111 (82.8%)	
	N.S. stated	19 (15.2%)	22 (18.3%)	13 (9.7%)	
Feeding tube at failure	Yes	83 (66.4%)	39 (32.5%)	40 (29.9%)	<.0001
	No	42 (33.6%)	81 (67.5%)	94 (70.2%)	
Tracheostomy at failure	Yes	54 (43.2%)	20 (16.7%)	17 (12.7%)	<.0001
	No	71 (56.8%)	100 (83.3%)	117 (87.3%)	

1 3D-CRT  =  Three dimensional conformal radiotherapy.

2 IMRTseq  =  Sequential intensity modulated radiotherapy.

3 IMRT+SIB  =  Intensity modulated radiotherapy with simultaneous integrated boost.

4 RT  =  radiotherapy.

On multivariate analysis (Table 4), Grade ≥3 mucositis was inversely associated with IMRT+SIB treatments (HR 0.15; 95% CI 0.04–0.51; *P* = .002), IMRTseq treatments (HR 0.29; 95% CI 0.09–0.87; *P* = .03) and laryngeal primaries (HR 0.33; 95% CI 0.14–0.78; *P* = .01). Grade ≥3 dermatitis was inversely associated with IMRT+SIB (HR 0.11; 95% CI 0.02–0.49; *P* = .004) and Platin-based chemoradiation (HR 0.10; 95% CI 0.03–0.27; *P*<.0001) and was directly associated with hypopharyngeal primaries (HR 4.08; 95% CI 1.20–15.10; *P* = .02). Less short term and long term feeding tube use was associated with IMRTseq as well as IMRT+SIB. Long term tracheostomy use was not significantly associated with any radiotherapy technique. When only patients treated with either IMRT-based planning were analyzed, IMRT+SIB remained predictive for less Grade ≥3 dermatitis (HR 0.23; 95% CI 0.06–0.50; *P* = .02).

**Table 4:** Multivariate analysis of toxicity (n = 379).

		Odds Ratio				
Timing of toxicity:		Acute toxicity			Late toxicity	
Category	Statistic	≥ Grade 3 mucositis	≥ Grade 3 dermatitis	Feeding tube during RT^1^	Feeding tube	Trach
IMRTseq^2^ (vs. 3D-CRT^3^)		0.29	0.47	0.20	0.15	0.66
	(95% CI^4^)	(0.09–0.87)	(0.14–1.52)	(0.06–0.60)	(0.04–0.42)	(0.20–2.23)
	*P* value	.03	.21	.003	.0002	.50
IMR+SIB^5^ (vs. 3D-CRT)		0.15	0.11	0.18	0.17	0.78
	(95% CI^4^)	(0.04–0.51)	(0.02–0.49)	(0.05–0.58)	(0.05–0.54)	(0.19–3.25)
	*P* value	.002	.004	.003	.002	.73
Platin-based regimen		0.72	0.10	1.28	0.74	0.47
	(95% CI^4^)	(0.36–1.44)	(0.03–0.27)	(0.68–2.46)	(0.40–1.36)	(0.21–1.05)
	*P* value	.35	<.0001	.45	.33	.07
Primary (OC^6^ referant)	Nasopharynx	0.74	2.36	0.33	0.54	2.20×10^−7^
	(95% CI)	(0.12–3.98)	(0.18–24.68)	(0.06–1.76)	(0.09–2.80)	(0–1.44)
	*P* value	.73	.50	.19	.47	.09
	Oropharynx	0.77	1.18	1.17	1.33	1.74
	(95% CI)	(0.37–1.62)	(0.46–3.02)	(0.57–2.42)	(0.67–2.67)	(0.72–4.43)
	*P* value	.49	.73	.66	.42	.22
	Larynx	0.33	1.32	0.57	1.05	8.40
	(95% CI)	(0.14–0.78)	(0.48–3.72)	(0.25–1.27)	(0.48–2.31)	(3.21–23.75)
	*P* value	.01	.61	.17	.90	<.0001
	Hypopharynx	0.46	4.08	1.26	1.47	7.84
	(95% CI)	(0.16–1.29)	(1.20–15.10)	(0.45–3.75)	(0.57–3.90)	(2.55–25.91)
	*P* value	.14	.02	.66	.42	.0003
	Unknown	0.30	6.40	0.56	0.37	0.96
	(95% CI)	(0.04–1.58)	(0.92–39.19)	(0.11–2.77)	(0.05–1.96)	(0.04–8.47)
	*P* value	.16	.06	.48	.25	.97
	Other	0.32	0.25	0.35	0.80	0.47
	(95% CI)	(0.08–1.06)	(0.03–1.36)	(0.10–1.12)	(0.24–2.57)	(0.06–2.41)
	*P* value	.06	.11	.08	.71	.38
≥2 drinks per day		0.55	1.04	1.71	1.18	1.56
	(95% CI)	(0.28–1.08)	(0.43–2.53)	(0.92–3.17)	(0.64–2.20)	(0.69–3.67)
	*P* value	.08	.93	.09	.60	.29
>10 pack-years		0.90	1.79	1.29	2.14	0.82
	(95% CI)	(0.37–2.21)	(0.53–6.57)	(0.55–3.04)	(0.89–5.46)	(0.25–2.99)
	*P* value	.81	.35	.56	.09	.75
Induction CTX^7^		1.53	1.43	1.25	1.23	0.62
	(95% CI)	(0.83–2.83)	(0.66–3.09)	(0.70–2.26)	(0.70–2.16)	(0.30–1.25)
	*P* value	.18	.36	.45	.48	.18
CTX dose modification		0.74	0.70	0.90	0.76	0.83
	(95% CI)	(0.40–1.39)	(0.30–1.58)	(0.51–1.60)	(0.43–1.33)	(0.40–1.71)
	*P* value	.35	.39	.73	.33	.62
Altered RT course	Delayed	1.19	0.48	1.23	1.10	1.59
	(95% CI)	(0.61–2.31)	(0.20–1.15)	(0.66–2.30)	(0.61–2.00)	(0.76–3.36)
	*P* value	.62	.10	.52	0.75	0.22
	Truncated	0.75	0.48	0.54	0.61	0.36
	(95% CI)	(0.31–1.77)	(0.15–1.36)	(0.24–1.20)	(0.27–1.33)	(0.11–1.03)
	*P* value	.52	.17	.13	.22	.06
Era of RT	2001–2006	2.68	0.96	0.88	2.96	0.41
(vs. 1993–2000)	(95% CI)	(0.87–9.15)	(0.29–3.17)	(0.26–3.27)	(0.99–9.97)	(0.12–1.31)
	*P* value	.09	.94	.84	0.05	0.13
	2007–2012	2.77	0.34	0.52	1.52	0.10
	(95% CI)	(0.71–11.82)	(0.42–13.11)	(0.13–2.29)	(0.42–6.02)	(0.02–0.48)
	*P* value	.14	.34	.38	.53	.003

1 RT  =  radiotherapy

2 IMRTseq  =  Sequential intensity modulated radiotherapy.

3 3D-CRT  =  Three dimensional conformal radiotherapy.

4 CI  =  Confidence Interval.

5 IMRT+SIB  =  Intensity modulated radiotherapy with simultaneous integrated boost.

6 OC  =  Oral Cavity.

7 CTX  =  Chemotherapy.

## Discussion

In our experience, IMRT+SIB provided outcomes similar to the traditional radiotherapy techniques for head and neck cancer. Here, two similar SIB schemes were used to treat 82% of patients indicating that our observations extend to commonly used IMRT+SIB plans. Compared to 3D-CRT and IMRTseq, we report similar disease control and potentially less toxicity in patients treated with IMRT+SIB. These outcomes with IMRT+SIB occurred despite the higher total doses delivered to the gross tumor in patients treated with either IMRTseq or 3D-CRT. In addition, compared to 3D-CRT, IMRT+SIB had lower rates of Grade 3 or greater mucositis and dermatitis as well as less feeding tube use during radiotherapy as well as long term feeding tube use even when the elective nodal areas received lower fractional radiation doses with 3D-CRT. By contrast, IMRTseq-based treatments were associated with lower rates of mucositis mucositis compared to 3D-CRT and higher rates of dermatitis when compared to IMRT+SIB. Therefore, our results suggest that IMRT+SIB may be as effective as other treatment strategies for locally advanced HNSCC.

In our series, we did not observe any differences in local or regional control for IMRT+SIB. These locoregional control rates are similar to other series examining IMRT+SIB where locoregional control ranged from 74–88% [8]–[11], [18]. It is important to note that many of these reports with improved locoregional control that analyzed patients with oropharynx cancers that may be due to HPV-positive disease. Since 2009 when we implemented HPV testing, only 7.8% of the 103 cancers tested were positive for HPV as measured by p16 immunohistochemistry. Therefore, even without a significant proportion of HPV-positive cancers, our outcomes were similar to the outcomes reported for series examining IMRT+SIB, IMRTseq or 3D-CRT. Furthermore, the outcomes for IMRT+SIB, IMRTseq or 3D-CRT were not dependent on the timeframe of radiotherapy as the locoregional control in patients treated between 1993-2000 was similar to patients treated between 2001to 2006 (HR 0.80; 95% CI 0.49–1.28; *P* = .35) and was similar to patients treated between 2007 to 2012 (HR 0.75; 95% CI 0.46–1.20; *P* = .23). Interestingly, we observed that increased dose per fraction using an IMRT+SIB regimen did not improve outcomes with locoregional control. Our data suggest that a slightly increased fraction size and shorter treatment time did not translate into clinically apparent differences between treatment outcomes. These results also parallel those of RTOG 0129 where altered fractionation was not superior to conventional radiotherapy when concomitant chemotherapy was given [19]. Therefore, the benefit of IMRT+SIB may occur with decreasing potential toxicity and efficiency in treatment planning.

In our series, IMRT+SIB lessened acute dermatitis compared to IMRTseq and lessened mucositis, dermatitis and feeding tube use compared to 3D-CRT. These differences in toxicity remained significant for 3D-CRT even when accounting for differences in primary sites, concurrent chemotherapy regimens and distinct timeframes of radiotherapy as well as other factors known to increase toxicity. Our rates of Grade 3 or greater mucositis parallels the 15% to 37.8% mucositis rates observed in other series reporting outcomes with IMRT+SIB [18], [20]–[22]. While IMRT+SIB may deliver higher doses per fraction to mucosal tumors that potentiate mucositis, lower fractional doses to other uninvolved mucosal sites may also minimize this toxicity. In addition, lower fractional doses to at risk nodal areas may also reduce the chances of dermatitis given the proximity of these regions to the skin. Finally, increasing the conformality of high dose regions may also minimize the long term dysphagia and tracheal toxicities as measured by long term feeding tubes and tracheostomy use. Therefore, despite the areas receiving higher fractional doses, IMRT+SIB may minimize both acute and late toxicities compared to IMRTseq and 3D-CRT.

While we observed less acute toxicity as measured by mucositis and dermatitis, we did observe increased higher rates of concurrent chemotherapy dose modifications for patients treated with IMRT+SIB. However, when accounting for other confounding factors such as concurrent-chemoradiation regimens, total doses to the gross tumor and induction chemotherapy use, IMRT+SIB was not associated with increased chemotherapy dose modifications compared to IMRTseq alone (HR 1.49; 95% CI: 0.89–2.51; P = .13). Therefore, IMRT+SIB did not likely impact chemotherapy dose modifications, radiotherapy treatment delays or truncations in the course of radiotherapy.

Our observations are limited due to the retrospective nature of this study. First, we reported on patients possessing heterogeneous clinical and treatment characteristics that may impact our observations of decreased toxicity in the IMRT+SIB cohort. Still, our observations of decreased toxicity with IMRT+SIB remained significant even when these factors were accounted for on multivariate analysis. Furthermore, we observed less acute toxicity with IMRT+SIB despite this group having the most use of concurrent chemotherapy that usually doubles the rates of acute toxicity. Second, our patients were treated with heterogeneous dosing regimens where patients treated with IMRT+SIB generally received lower total doses to the gross tumor and higher total doses to the intermediate and low risk nodal levels. Nevertheless, these dosing strategies are, in part, linked to the treatment technique indicating that doses delivered by an IMRT+SIB technique provided, at worst, similar disease control and toxicity. Given that our treatment deliveries were verified using standard megavoltage portal imaging, our PTV margins remained similar for each treatment technique. Therefore, our observations were not impacted by image guidance techniques that enabled smaller margins. In addition, our patients were treated from 1993 to 2012 during which many changes in the treatment of HNSCC patients occurred. Nevertheless, our conclusions regarding less toxicities with IMRT+SIB hold even when accounting for these distinct eras of treatment on multivariate analysis. Finally, our data likely extrapolates to patients with HPV-positive cancers in terms of efficacy as well as toxicity as only a fraction of our patients tested positive for HPV by p16 IHC. Therefore, when accounting for confounding factors, we observed that IMRT+SIB likely predicted for similar, if not better, outcomes compared to traditional treatment strategies.

Comparing to traditional treatment techniques such as 3D-CRT and IMRTseq, we find that IMRT+SIB was an acceptable alternative as assessed by disease control and toxicity. This technique enables more efficient treatment planning with less uncertainty as well as potentially shorter treatment times. Furthermore, IMRT+SIB may afford even less acute and late toxicity potentially through less fractional doses to the elective nodes. These differences in toxicity await validation in future randomized control trials.

In conclusion, we find that IMRT+SIB provided similar outcomes in terms of disease control and toxicity compared to 3D-CRT and IMRTseq. IMRT+SIB delivered lower fractional doses to the elective nodes and higher fractional doses to the gross disease. Furthermore, it is likely that these results extend to both HPV-positive and HPV-negative patients. These results support the use of IMRT+SIB as an acceptable technique to treat patients in order to more efficiently plan radiotherapy while maintaining similar outcomes as in previous techniques.
